# Summer Research Internships Prepare High School Students for 21st Century Biomedical Careers

**DOI:** 10.15695/jstem/v4i1.13

**Published:** 2021-11-29

**Authors:** Anushka Patel, Arlene Bulger, Kevin Jarrett, Shirley Ginwright, Katie Busch Chandran, J. Michael Wyss

**Affiliations:** 1 College of Arts and Sciences, Center for Community Outreach Development (CORD), The University of Alabama at Birmingham, Birmingham, AL; 2 Departments of Cell, Developmental and Integrative Biology and Medicine, The University of Alabama at Birmingham, Birmingham, AL

**Keywords:** STEM, internship, mentor, assessment, lead researcher, biomedical careers, underrepresented minority

## Abstract

STEM internships for both high school and college students provide early opportunities for students to discover careers of interest and career paths they may not otherwise experience. For over 25 years, the University of Alabama at Birmingham’s (UAB) Center for Community OutReach Development (CORD) has provided rising high school seniors with opportunities to conduct research in federally-funded laboratories under the mentorship of UAB faculty. This paper evaluates CORD’s High School Summer Science Institute III Program (SSI III) and its impact on participants’ STEM career trajectories. Outcomes were tracked for SSI III participants over an eight-year period, and former interns’ perceptions of the program reported. Over 99% of surveyed interns (N=102) chose a STEM undergraduate major, and 97% of the former interns reported they were pursuing STEM careers. Nearly all interns indicated their SSI-III experience was very positive and influenced their career decision. Over half of the interns matriculated into an undergraduate STEM major at UAB, providing the university with return as more excellent students for their investment in the program. These results highlight the importance of high school student involvement in STEM internships as a pathway that leads towards STEM careers.

## INTRODUCTION

The demand for Science, Technology, Engineering, and Mathematics (STEM) professionals has rapidly increased, as the US and other countries recognize the need for a STEM-trained workforce, see, e.g., (Bautista et al., 2018; National Academies of Sciences and Medicine, 2016; National Acadmeny of Sciences and Engineering, 2010). Further, traditional “non-STEM” jobs increasingly require skills in STEM areas that go well beyond basics, like knowing how to use Microsoft Word (Jang, 2016). For instance a assembly worker at the Mercedes Benz automobile plant in Alabama needs to operate robots in their job. Yet, the United States (US) continues to have an education system that turns out graduates with average science and math capabilities, ranking the US in the middle of the 71 developed countries with whom the US competes (Kastberg et al., 2016). Further, while women have made significant gains in the STEM workforce, Hispanics and Blacks remain greatly underrepresented.

The underrepresentation of Black, Hispanic, and Native Americans is a well-documented disparity in STEM careers (Estrada et al., 2018; Hurtado et al., 2010; Stockard et al., 2021), and many historically underrepresented minority students feel more challenged and have a lower sense of belonging in STEM careers. Therefore these students continue to, struggle to see themselves integrating and flourishing in these careers (Aish et al., 2017). Countless initiatives in the US strive to find effective ways to populate the STEM representation gap among these minorities, but there remains a long road ahead in resolving this disparity. Research has shown that having near-peer role models (Aish et al., 2017), quality mentors, research experiences (Estrada et al., 2018), and high-level science coursework (Hurtado et al., 2010) can help individuals from populations underrepresented in STEM careers (URM; in these internships this group included Blacks and Latinx students) feel prepared for and give them a sense of belonging in undergraduate STEM programs and careers. University internships can also assist students financially and ease their transition to a four-year institution since they will already have supportive connections at the four-year institution that can help facilitate the URM student’s success in completing STEM degrees (Hurtado et al., 2010; Jackson et al., 2013; Stockard et al., 2021). Our internship program focused on students from diverse backgrounds.

To solve the complex problems of today’s world, graduates will need STEM knowledge and skills to understand complicated challenges, analyze, and master them. Improved STEM education will help develop the next generation of critical thinkers, increase scientific knowledge, and empower innovators. The first part of the 21st century witnessed a decline in the number of high school students choosing STEM fields (Chen, 2009). One method to overcome this lack of engagement in STEM careers is to provide research experiences in which research mentors engage students in important research projects. These opportunities have begun to emerge at research-intensive US institutions of higher education and in businesses.

The University of Alabama at Birmingham’s Center for Community OutReach and Development (CORD) has offered high school students an in-depth opportunity to engage in STEM fields through its Summer Science Institute (SSI), which is divided into three specific programs, SSI-I, SSI-II, and SSI-III (Chandran et al., 2020). In SSI-I, high school students learn the fundamentals of molecular and cellular biology. In SSI-II students focus on advanced principles of molecular biology and neurobiology. The content and skills, in both SSI-I and SSI-II, are taught through various training activities, group discussions, and lab experiences in intense two-week programs. SSI III students first receive training in molecular biology techniques, after which.each student is paired with a funded UAB researcher, who mentors the student throughout their summer internship experience. The interns spend eight weeks conducting biomedical research on a specific hypothesis that they develop, and they present their research at a competitive poster symposium at the end of the summer. These programs are open to applicants from all socio-economic backgrounds, and scholarships are available to students who demonstrate financial need, thus eleminating financial barriers of participation. In the current study, we evaluated the impact of these research internships (SSI III) on STEM education and the interns’ career trajectories.

## METHODS

### Program Overview.

The SSI III summer research internship is open to rising high school seniors. Participation in the program includes an intensive 40 hour per week, eight-week internship. Recruitment of students for the program was four-fold: 1) recruiting the students through SSI I and II (most interns came via this pathway); 2) mailings/e-mailings to high school STEM teachers in Alabama; 3) CORD’s website postings; and 4) recruitment from participants in CORD’s Regional Science and Engineering Fair.

Prior to joining an assigned mentor’s lab, the interns completed an intensive 2-day orientation in which they were trained in biomedical laboratory techniques, building on the knowledge and skills previously learned in other CORD programs (Chandran et al., 2020). The students were also required to complete formal training in the UAB research code of conduct and privacy, use of human subjects, laboratory safety, and the care and use of animals in research. Each intern was then assigned to a UAB faculty research mentor under whom they developed an independent research project related to the mentor’s research focus. The mentor assignments were made by the SSI directors based on each student’s career interests. Although students occasionally requested to be placed with a specific family friend or relative, such placements often proved not to be as productive as when the students were matched based on research interest alone.

Interns spent about 38 hours per week working in their research laboratories. They also attended workshops and scientific seminars, and toured UAB research facilities as a group during their remaing time on campus. At the end of the internship, each intern exhibited their original research findings as a poster presentation during the CORD closing ceremony. Mentors and other faculty were invited to view the posters, allowing the interns the opportunity to discuss their research findings to members of the scientific community. Their work was evaluated with similar criteria to that used for presentations made by undergraduate summer research interns at UAB, and they competed for awards including a scholarship to UAB. Participants were encouraged to enter their research in the UAB CORD Regional Science Fair the following March, in which they competed for prizes at the regional, state, and international levels. Research interns were paid a stipend for their participation in the program including the two days of training ($1,000-$2,000, depending on the year).

### Student Selection and Demographics of Participants.

Selections for research internships were made by an admissions committee, which evaluated the applicants’ high school academic performances, their interest in science (evidenced by their essay), standardized test scores, previous SSI experiences, and interviews. Students had to meet the following inclusion criteria to be selected for an internship: U.S. Citizen or Permanent Resident, a rising high school senior, and have an expressed interest in a STEM-related career. Applicants were also required to provide two letters of recommendation (at least one from a science teacher), a one-page essay describing their personal, and career interest in a STEM career, and commitment to a full-time (40 hours per week), eight-week summer research internship.

After reviewing applications and conducting interviews, the students were admitted based on their qualifications for the available number of internship slots (~13/year). Priority was given to students who had attended SSI-I and/or SSI-II, as well as to those who demonstrated a passion for research and who might not otherwise have access to an opportunity like the SSI-III program. About 90% of all students accepted the internship and agreed to the strict requirement for no more than two days of excused absences during the 8-week period. Students who declined internship offers typically based their decision on family or other organizational commitments that could not be altered (e.g., foreign travel with their family, sports camps, etc.).

For the research reported here, 102 high school students were assessed from the SSI-III cohorts from the summers of 2012 to 2019. Most applicants had pre-existing interests in a STEM-related career, influenced by various factors including family, teachers, peers, and initial career interests. Approximately 90% of the interns were from local public and private schools (i.e., within 80 miles of Birmingham). From 2012–2019, an average of 67% of the summer research interns were Black, with annual percentages ranging from 55% to 83%. About 60% of all SSI-III students previously attended SSI-I and/or II.

### Faculty Mentors.

Mentors were recruited from faculty members in diverse scientific disciplines. Their primary UAB appointments were in Anesthesiology, Biochemistry and Molecular Genetics, Biology, Bioinformatics, Biomedical Engineering, Cardiovascular Medicine, Cell Developmental and Integrative Biology, Chemistry, Computer and Information Science, Civil and Environmental Engineering, Dentistry, Dermatology, Electrical, and Computer Engineering, Epidemiology, Genetics, Medicine, Neurobiology, Neurology, Nursing, Nutrition, Optometry, Pediatrics, Pharmacology/Toxicology, Physiological Psychology, Physics, Optics, Physical Medicine and Rehabilitation, Physics, Psychiatry, Psychology, Public Health and Behavioral, Neurobiology, Radiology, and Radiation Oncology, and approixatemely 70% of the mentors conducted biomedical research.

Mentors were recruited in mid-January, and each mentor completed an application including information on their area of research and confirming their interest in mentoring a CORD SSI III summer program student. Mentors were required to have adequate federal or equivalent extramural research funding, an active research laboratory, and publications documenting their recent research efforts. Each mentor was responsible for ensuring interns passed all institutional, state, and federal occupational health and safety regulatory training, and the responsible conduct of research modules.

The mentors were required to give the interns a realistic expectation for time management and commitment, independence, an understanding of their scientific discipline, and relevant skillsets. The mentors worked with the interns to develop a summer research project with well-defined expectations that were pursued under their supervision. Several faculty mentors with larger laboratory operations assigned their interns to a near-peer mentor, e.g., a postdoctoral fellow, a graduate student, or an experienced laboratory manager who oversaw the day-to-day conduct of the interns’ research.

### Pre-Internship Training.

Research interns were required to attend an intensive 2-day orientation at UAB, in which they reviewed or were trained in knowledge and skills learned in SSI I and SSI II. The orientation also included training in research compliance and safety, hands-on biomedical science technical training, protocol development, data analysis, and data interpretation. The interns completed in-person safety training and signed a safety contract agreeing to comply with the UAB Department of Environmental Health and Safety research safety guidelines. Interns completed additional safety training and training for the use of human or animal subjects, based on their mentor’s research.

During the hands-on laboratory training, the interns refreshed basic laboratory techniques in weights and measurements, pipetting, calculating percent solutions, reagent preparation, and other scientific calculations such as calulating molar concentrations. Participants trained in molecular biology techniques including: enzyme-linked immunosorbent assay (ELISA), gel electrophoresis, and polymerase chain reaction (PCR). These skill sets helped increase their confidence and encouraged independence in a research laboratory setting. The interns acquired much more specific research training while working on their independent research projects.

### Staff Visits to the Interns.

The program staff director visited each student weekly throughout their internships. These check-ins ensured that the interns were on track with their research and enabled the director to help them navigate and resolve any challenges encountered by the students or the mentors. Occasionally the SSI III director encountered a situation in which there was an obvious mismatch between the intern and the mentor (<1 intern per year), and once identified, the program director worked with the student and mentor to improve the situation or to change the intern’s laboratory. In these cases, the situation was always resolved to the satisfaction of all concerned. The director also queried the students about their work during the weekly workshops and in the labs to ensure that they were making appropriate progress. The program director, a former lab manager, acted as a liaison between research interns and intern mentors thus ensuring favorable intern outcomes.

### Final Presentations and Competition.

**Figure 1.**
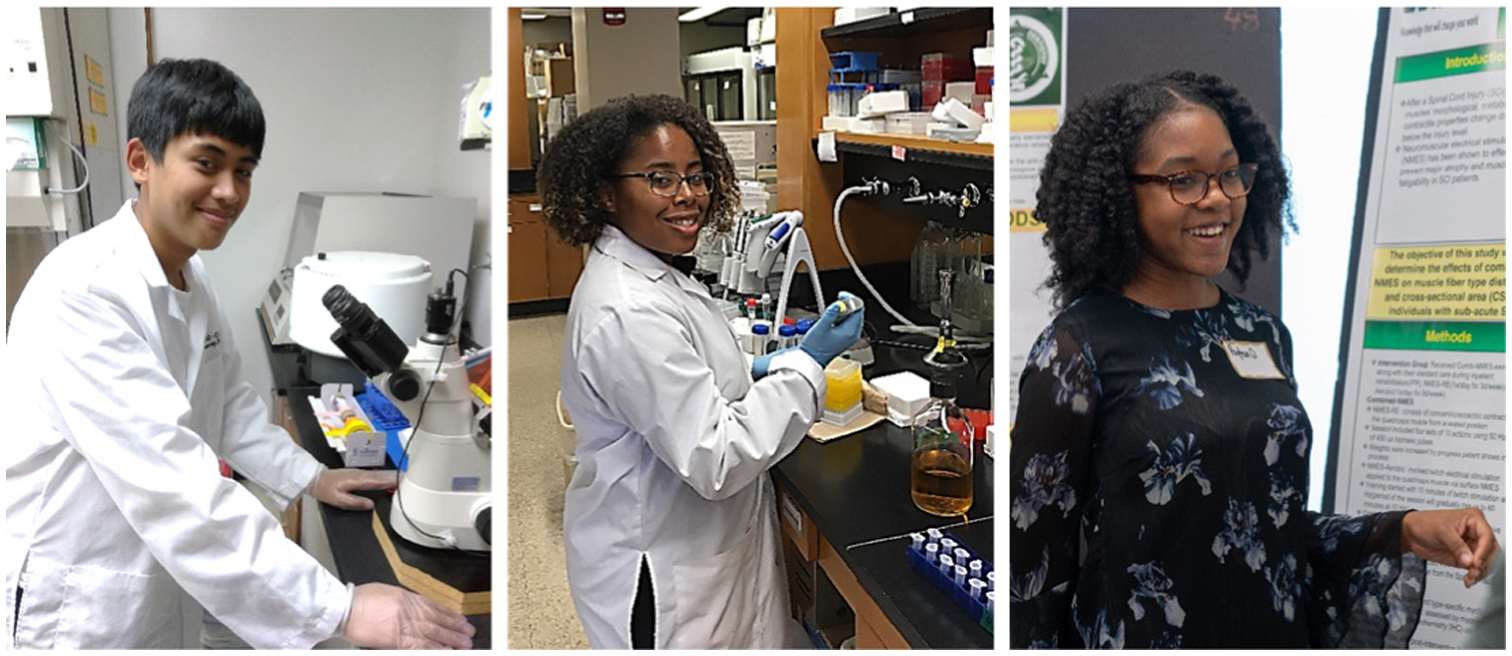
Pictured in the left and centered panels are Jacob Mesina (Neurobiology) and Paris Maddox (Molecular and Cellular Pathology) at work. The right panel shows Peyton Debrow presenting her research that won her the Vice President Student Affairs Scholarship.

At the conclusion of SSI III, interns were required to give an individual competitive poster presentation summarizing their summer research findings at the SSI Closing Ceremony (Figure 1). The students were given instructions at the weekly meetings about how to construct a good poster and given examples of such, and presented various aspects of their final presentation at the weekly meetings. Finally, all posters were critiqued by the director and the mentor prior to finalization and printing. UAB faculty, researchers and local, highly qualified STEM professionals (5/intern) interviewed each student and judged their research methods, findings, presentation, and conclusions to select winners based on their accuracy to convey research findings, scientific merit, and methodologies. All interns received a Certificate of Achievement for completing the internship program.

### Data Collection and Analysis.

In May of 2020, questionnaires were emailed to SSI III interns (2012–2019) to acquire information on demographics, secondary and college education, projected dates of graduation, if their degree was biomedical science or STEM-related, and if they felt their SSI III experience influenced their career decision.

An external evaluator assessed the performance of the SSI III interns, who were interns in the summer of 2019. The data were collected via email and queried in the following areas: the ability to assemble and present a persuasive research poster, ability to construct a report using formal scientific writing format, skills for preparing and presenting research topic to peers, ability to work independently and as a member of a team, problem-solving skills, commitment to graduating with a Bachelor of Science or Engineering degree, interest in a career in a STEM field, and interest in a career that includes research. The evaluation also assessed 1) the interns experience in being mentored by the “lead” researcher or by others (graduate students and postdoctoral fellows), 2) the climate of the research experience in terms of encouraging independence, 3) sharing of ideas, encouragement provided when needed, 4) appreciation for the work done, 5) the usefulness of lab tours of centers and facilities, and 6) the weekly Thursday seminars. Each area was rated by the interns on a scale of 1–5 (1-poor, 2-fair, 3-good, 4- very good, and 5-excellent).

The interactive workshops attended by the interns during the summer were designed to supplement the program relative to research safety and practices, shared knowledge, protocol development, statistical methods, problem-solving, and scientific communication techniques. Interns were required to attend the following weekly undergraduate research workshops; 1) undergraduate research 101, 2) lab safety/lab basics 101, 3) research ethics and professionalism, 4) writing a winning research abstract, 5) creating the perfect poster, and 6) giving a winning presentation. The quality of these workshops was rated by the interns on a scale of 1–5 (1-poor, 2-fair, 3-good, 4-very good, and 5-excellent).

The learning outcomes of the research experience were evaluated in relation to Bloom’s Taxonomy for the understanding of new material (Forehand, 2010). This assessed the interns’ ability to investigate or create new or original scientific research, examine or justify a decision, test hypotheses and analyze experiments, interpret and apply new information, understand and explain scientific theories, and remember facts and basic concepts. The interns rated their overall learning experience from five different choices: waste of time, did not learn a lot, so-so, learned a lot and this is a fantastic way to learn about research.

## RESULTS

**Figure 2.**
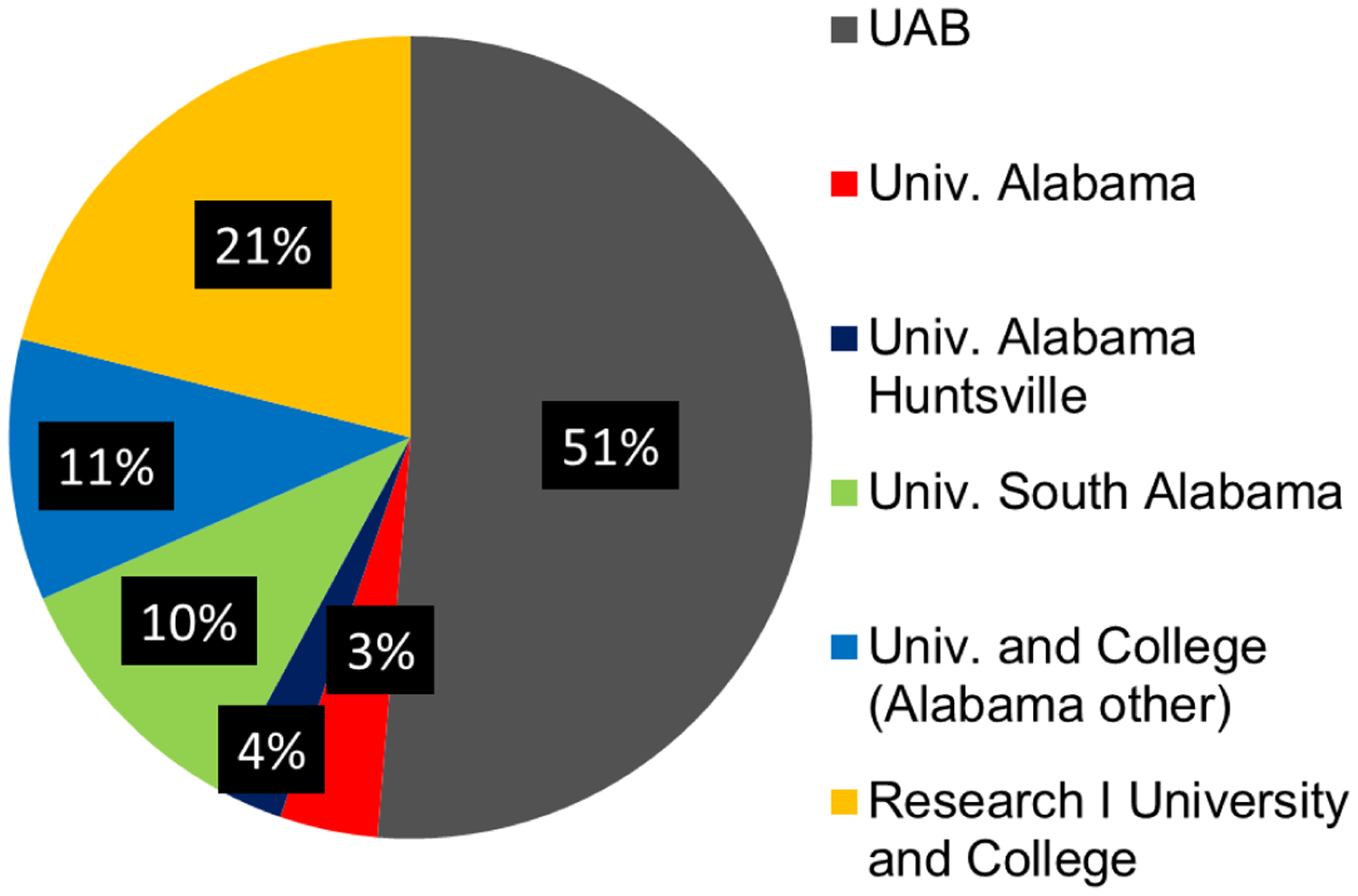
University and College Enrollment of UAB-CORD SSI III Interns, 2012–2019. A pie chart depicting the university and college choices made by the 2012–2019 SSI III research interns.

**Figure 3.**
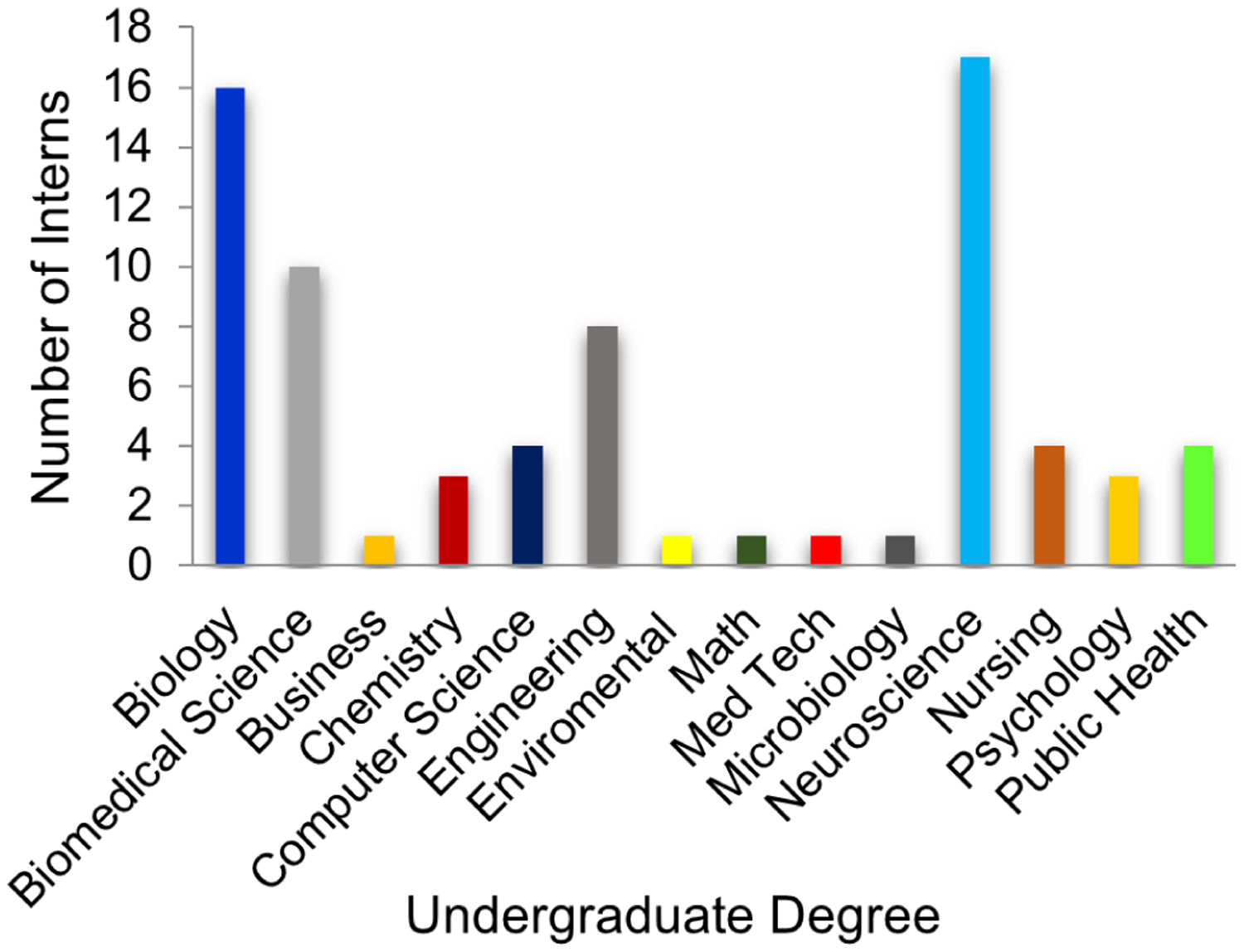
UAB-CORD Summer Science Institute III Interns. Initial Undergraduate Education, 2012–2019. The college majors being pursued by the 2012–2019 SSI III research interns.

Over the eight years that were evaluated in this study, all 102 SSI III interns who were accepted into the program completed the full internship and all interns competed in their year’s final poster competition. All 102 interns were tracked by previously obtained contact information, Facebook postings, LinkedIn, and similar resources. They were asked to complete a short REDCap survey about their internship experience and its impact on their careers (Harris, 2009). All but one of the 102 interns completed or are completing a bachelor’s degree in a STEM major within five years of high school graduation. The only intern who did not continue in a STEM career pathway completed a business degree. Relative to colleges/universities attended by the interns, the majority of interns attended UAB. Another 28% of the interns are attending/graduated from other Alabama colleges and universities (Figure 2). The remaining 21% of the interns went to out of state, mostly research 1 universities including Emory, Johns Hopkins, the US Military Academy at West Point, Vanderbilt, and Washington University in St. Louis. The most often chosen undergraduate majors of the students were biology and neuroscience, with biomedical science and engineering the next most popular (Figure 3). The initial career choices (including the intentions of those not yet graduated) were split between clinical practice and research, i.e., 42% of the interns chose a path to health care licensure (MD, DMD, OD), but many of these students are pursuing clinical research careers. All students responding indicated that the summer research internship was important in their career progression. Most of the interns (>90%) indicated that the research internship significantly influenced their career choices. Additionally, 62 of the 102 interns competed in the Central Alabama Science and Engineering Fair in March of their senior year, and 95% won a position to compete in the Alabama State Fair and several each year have won a position to compete at the International Science and Engineering Fair. Thus, the research the interns carried out was very competitive. Furthermore, most of the interns were authors on abstracts at national meetings that were based on their summer research.

### More Specific Outcomes Data from the 2019 Interns.

The survey of 2019 summer research interns found that 80% rated their principal mentor as very good to excellent, while 100% of the interns rated their near-peer mentors (graduate students and postdoctoral fellows) as very good to excellent. Interestingly, only 30% of the interns rated their principal mentor as excellent, while 70% of the interns rated their junior mentor(s) as excellent, likely due to the greater contact time between the interns and the junior (versus principal) mentors and the near-peer relationship of the near-peer mentors. Nearly all interns found the research environment of their lab to be good to excellent. The interns liked the tours of other research labs on the UAB campus, but they had more mixed attitudes toward the weekly seminars; 40% considered them poor to fair. In written comments, the interns indicated that they did not like losing the 1½ hours to the seminar each week. In regards to the overall research experience, 90% of the 2019 interns found the experience very good to excellent (i.e., they considered the internship an excellent learning experience).

Based on the interns’ self-assessments, their participation in the summer 2019 internship increased their confidence in communicating and presenting research, writing a research report using formal academic writing styles, and working as a member of a science team. They also felt increased commitment to graduate with a bachelor’s degree in a STEM major and were interested in pursuing a career that included research (Table 1). Effect sizes were calculated from a matched pair dependent t-statistic (Lenhard and Lenhard, 2016). Participation in the 2019 UAB-CORD SSI III program had a medium effect (d = 0.57) on increasing students’ problem-solving skills and a small effect (d = 0.44) on increasing students’ ability to work independently. Some of the largest effects were on “Your interest in a career in research” (d = 0.98) and “Your confidence in putting a research poster together” (d = 1.96).

### Qualitative Data.

The following are semirandomly chosen student responses to the following questions: “What were the most important positive things you got out of being part of the UAB-CORD SSI III program?” “Do you feel that your Summer Science Internship (SSI III) experience influenced your career decision?”

#### Most Important Positive Influences.

“Being able to work with people who are actually researching potential markers for neonatal brain injuries in infants was exciting.”

“I learned about different fields of science I can go into and how they overlap.”

“I made a strong relationship with the graduate student I worked with and with my mentor. I liked how everything was hands-on because that is different from my normal experiences of learning from a textbook in school. I also got to see cool cutting-edge research and how there were challenges because nobody else has explored an area before. Exploring the unknown is what appeals the most to me about research. It involves a lot of critical thinking that I got to see the process and participate in it as well.”

“I really enjoyed all the work and the experiments I got to do in the lab. I also thought that the Undergraduate Research Expo at the end of the summer was a great learning experience. I also enjoyed that I got a stipend after completing the internship.”

“I was able to work in a lab that had a lot of equipment that I had never seen or used before.”

#### SSI III Experience Influenced Your Career Decision.

“I think that SSI III opened my eyes to how important accuracy and thoroughness was in science. My internship pushed me to understand research and solidify my understanding of my work. SSI III introduced me to interactions between the scientific world and the physicians that use research findings to pursue their careers. I’m now considering incorporating research into my medical career.”

“I had the opportunity to conduct hands-on research with a well-respected UAB faculty professor. Through the program, I gained valuable research experience, learned laboratory techniques, and most importantly shared the passion of science with a professional in the field. This experience helped me solidify my interest in pursuing research opportunities during my undergraduate career.”

“My time in SSI III influenced my career decisions. As an intern, I gained a lot of experience working in a lab setting that I would not have been able to have as a college freshman. I learned not only how to prioritize my time, but, I also learned how to use the different equipment in the lab and how to create a research project. After my internship, I knew that I would be comfortable and happy working in a research environment again.”

“Definitely, the recommendations from my mentors at CORD were the whole reason I learned about Early Medical Admissions Program and the UAB School of Medicine, and it played a tremendous role in getting me into those programs. Furthermore, CORD was my first exposure to clinical research, and it solidified my interest in working in the area as a career. If I were exposed to a typical high school research experience of doing rote bench work with no real input in the process (instead of being in CORD SSI III), I probably would not have pursued this career choice”

## DISCUSSION

The current assessment indicates that CORD’s high school summer research internships had a positive effect on the STEM education and research career trajectories of the participants. The interns indicated that the internship increased their confidence in reading and understanding primary scientific articles, thus preparing them for the rigors of college STEM classes (Chiappinelli et al., 2016). Furthermore, the internships had an impact on the career trajectories of the interns (Papadimitriou, 2014). Many of the interns came to the SSI III program with the vision of getting their “card punched” for admittance into programs leading to clinical training in a health career. We expected that many students interested in science would aspire to a clinical career, since physicians, dentists, nurses, optometrists, etc., are the primary, if not only science professionals with whom the school-age children (especially those from underserved communities) come into contact. The pre-/post- survey data demonstrated that the hands-on experiences in the internship gave the students an increased confidence in their problem solving skills related to science (Table 1). Participants from populations historically underrepresented in research careers often do not know anyone who is a scientist and thus lack scientist role models to follow. By providing such role models, the internships make these participants aware of the potential excitement and rewards of a research career other than the ones they observe in clinicial professions (Kabacoff et al., 2013). We note that 21% of the mentors were minority, and 67% of the CORD staff running the program were minority faculty and staff.

In this regard, it is not surprising that the interns felt closer to, and slightly better mentored by, their near-pear mentors than by their PI mentors (Tenenbaum et al., 2014). Interns assigned to large or medium-sized laboratories were typically assigned to a graduate student or postdoctoral fellow in that lab, which typically gave the graduate students or fellow excellent training in mentoring. While the PI mentors met regularly with their SSI intern, the near-peer mentors were the ones who worked with the interns throughout each day. Further, the near-peer mentors were typically closer in age to the interns, giving many of the interns the realization that they could be a researcher, just like their near-peer mentors. Often it is more difficult for interns to realize they could be like their PI, when the PI is two decades or more older than the intern (Tenenbaum et al., 2014).

Summer STEM internships also appear to increase students’ college readiness in STEM disciplines (Kallison, Jr and Stader, 2012). Often this is related to the hands-on activities of the internship, which are especially effective in exciting students about research careers. The interns also gain significant writing and analytic experience, which improved their ability to navigate sometimes difficult undergraduate courses. Similar to the experience of other internship programs in colleges, the SSI interns and mentors indicated that one of the greatest advantages of research internships was giving students the opportunities to apply knowledge and skills to real-world problems in a real-world workplace (Brush et al., 2014). The SSI internship includes training in reflective writing throughout the internship. This leads the interns to a deeper understanding of the hands’-on research that they conduct (Minnes et al., 2017). Also included in SSI III, is a focus on analytic methods, especially statistical analysis in the design and interpretation of research. Few of the high school students and or even undergraduate students have more than a rudimentary understanding of statistical methods, yet these are vital parts of understanding and interpreting scientific design and the resulting data. Thus, the weekly workshops included sessions in statistical design and application, which were also stressed as the students presented their research designs and findings. This training was useful for all of the interns but especially for the students from high needs schools who often need mentoring that includes strengthening basic academic skills, increasing professional skills and reinforcing an attitude of “I can do this” (Kabacoff et al., 2013). The students also develop a strong constructivist learning outlook, helping them to think more creatively, independent of teachers’ lectures and textbooks (Hsu and Espinoza, 2018).

**Figure 4.**
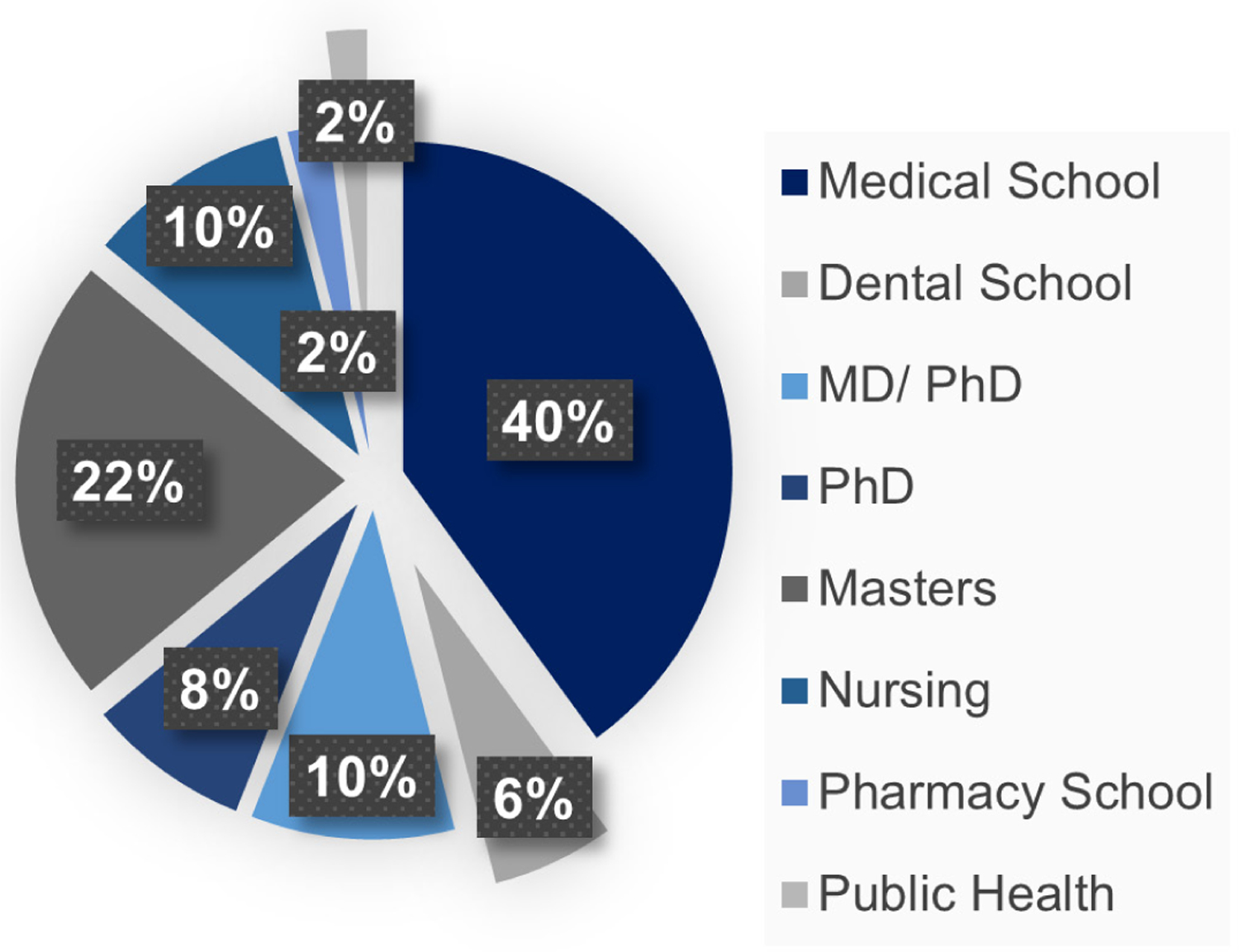
UAB-CORD Summer Science Institute III Interns, 2012–2019. Initial careers and higher education goals of UAB-CORD interns.

**Figure 5.**
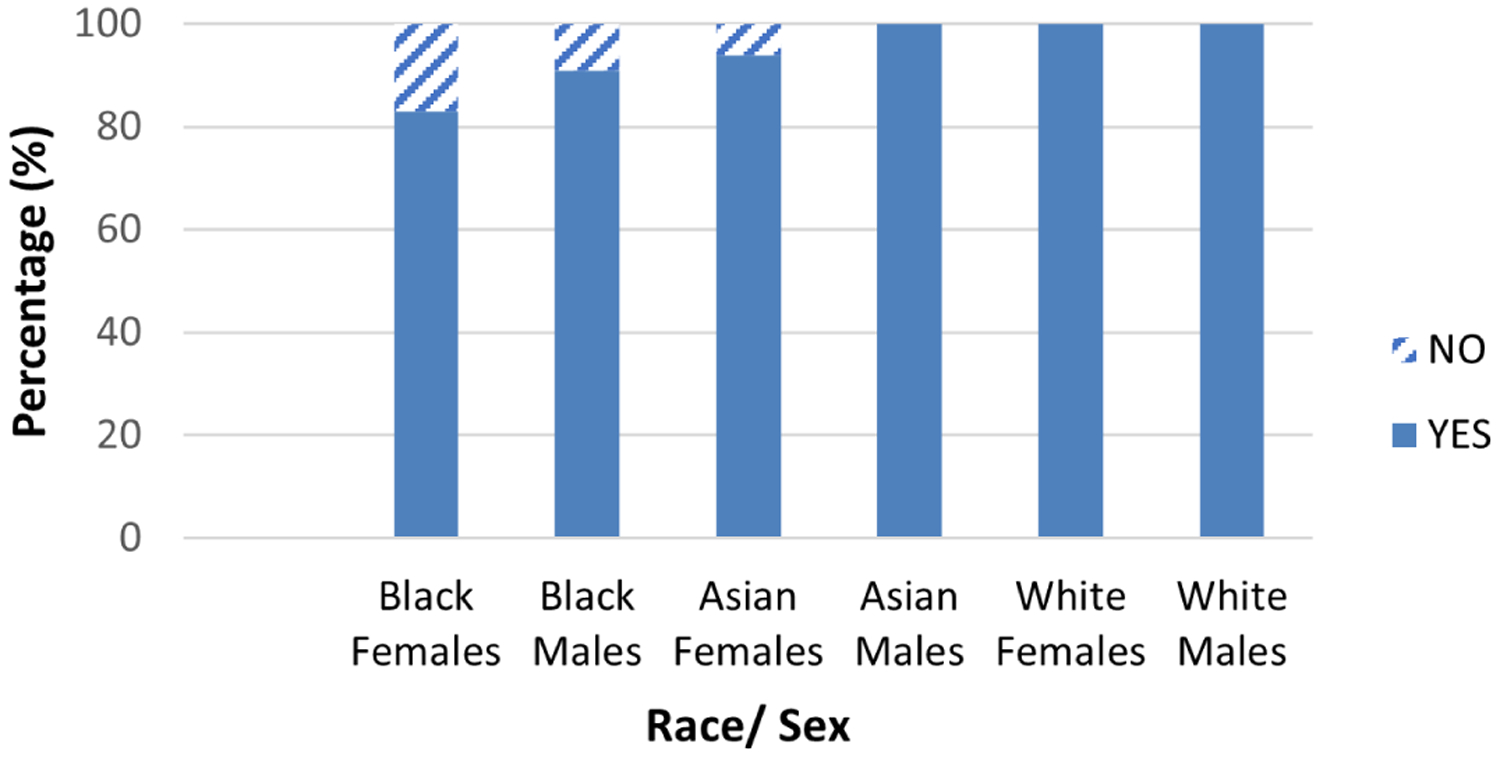
Program Outcome: Do You Feel That Your Summer Science Internship Influenced Your Career Decision? 2012–2019 interns perception of how much the SSI III influenced their initial career choice.

Thus, an early authentic research experience in STEM fields of interest provided the SSI interns with the opportunity for hands-on experiences, giving them content knowledge and skills, an understanding of scientific concepts and data analysis, and communication skills that they used to present their findings at local symposia and national conferences, leading to their successful transition to college STEM pathways. UAB received the largest percentage of 2012–2019 SSI III interns as undergraduate students (Figure 2). This likely results from professional networking during the internships, including insights gained at STEM seminars, communication with fellow interns about their research, campus familiarity, and the opportunity to continue research with their mentor as an undergraduate. The SSI III graduates primarily selected biology, biomedical science, engineering, and neuroscience (Figure 3) as their majors. This indicates the interns’ interest in studying in STEM fields. Nearly all interns plan to continue their education to an advanced STEM degree (Figure 4), and the internship experience helped influence their career decision (Figure 5). What is interesting in this regard is that relatively few of the interns (<40%) indicated an interest in a research career at the start of the internship, with most indicating that they were going to pursue a clinical career. However, by the end of the internships, the majority of participants were interested in clinical research or exclusively research career. Program participation increased students’ feeling of self-efficacy in laboratory research, thus increasing their interest in a STEM career and solidifying the desire of students with a pre-existing interest for such a career. We note that there is a bias in our data relative to the selection of the students for internship. The students had to have a strong interest and understanding of science to be accepted into the program. However, when we queried the students at the start of each internship year, over 50% were interested in a purely clinical career. This is not unexpected since few high school students are provided an experience that demonstrates to them the excitement of research and experimental discovery.

Most of the interns were interested in biomedical sciences and engineering, and believed that it was important for them to work in a research laboratory and build relationships with lab members, thus experiencing team science. The assessment also indicated that participation in SSI III workshops, seminars, and the research experience had a positive effect on increasing the interns’ confidence in working independently, and the ability to be a team member, communicate research to their peers and researchers, and have the information needed to choose if a STEM undergraduate major and a STEM career was right for them. Interestingly, the exposure of the interns to experienced teaching mentors has led a few to explore secondary and/or college STEM teaching careers (Borgerding, 2015).

Over the course of the past 25 years, CORD has learned much about effective summer internship research programs. First, to be able to recruit diverse and well-prepared students into the internships, the program needs a pathway that extends to lower grades, including high school but also K-8 grades. It is at the lower grades that the initial flame is lit in the students to consider pursuing a STEM career (Chandran et al., 2020). Second, it is important to have adequate funding and to have engaged faculty mentors who are willing to provide excellent training and supplies to assist the students in their research discovery. Third, finding the right individual with outstanding research experience to follow the students’ progress can help the interns navigate research and quickly resolve issues and personal problems that can seem overwhelming to the high school students in a strange new environment. Fourth, if one is to recruit underrepresented groups into internships, it is important to pay them. Taking a summer job helps the students alleviate family financial burdens, and the internship requires a large time commitment that is not conducive to holding a second job. Fifth, we feel that it is important to help the students learn a strong work ethic in their internship. We allow only two excused absences for the program, typically for campus tours at other universities or sick leave. The student is responsible for requesting the time off and having it approved. We have not had any student since 2009 who failed to comply. A final lesson learned was the need for early acceptance decisions and very quick enrollment of the interns into the university’s administrative system before they started their internship. This enables them to log in to training requested by mentors, e.g., IRB, IACUC, Occupational Health, etc. Having the required training completed before starting the internship ensured the interns could begin their research projects in a timely manner.

Internships are an important part of a student’s resume. They can be beneficial for entrance into undergraduate programs and scholarships, and entrance into graduate school and other professional programs. Students learn to integrate theory and knowledge learned in the classroom with real-world application and skill development in a research setting. Training, skills, and networking can be an asset for future employment. The pivotal experience of an internship can greatly assist a student in defining his/her career goals.

### Funding.

SSI III was initially funded by Howard Hughes Medical Institute, which supported $2,000 stipends to each intern and $1,500 for each mentor. When that funding ended in 2002, a private donor (Alabama Scholars) continued to fully fund only the student stipends. Fortunately, our very collaborative mentors did not object to conducting the internship training without receiving lab financial support. In 2014, our donor ceased funding the program and it has been supported by CORD reserves and program revenues since then. Currently, the SSI III interns receive $1,000 for the internship. Notably, this lower stipend has not greatly decreased the number of applicants, but it has decreased the percentage of students applying from populations historically underrepresented in STEM careers.

There are several important aspects of funding for a successful research internship program. The main element that makes an internship program work is the resources supplied by the research mentors, including their time, staff time, laboratory resources, and supplies that are needed to conduct the research that the interns will carry out. When funding for the program shifted, we found that the lack of financial support for the lab did not diminish the enthusiasm of the research mentors. Each year new faculty members volunteer to mentor enrolled high school students. The mentors’ resources of supplies, equipment, and time dwarf all other funding.

A second element making the internships financially effective is the funding of the interns’ stipends. When we have asked students, who completed SSI I and II why they did not apply for a summer internship, the response is generally that they needed to make more money in the summer. CORD required that interns did not work a second job and that they carry out their assigned research and training for 40 hours per week for the full 8 weeks of the internship. Thus, the lower stipend amount that resulted from a shift in funding was a significant impediment for many of the students we most desired to reach. This does not appear to be the result of these students losing interest in research careers. In a similar summer research internship program that CORD offers to community college students, the applicant numbers continue to increase each year for minority students, but in that case, the interns receive larger funding for their summer research efforts. While a few federal agencies will provide relatively long-term funding for these programs, most grant mechanisms are for shorter terms (3–5 years), and thus a sustainable program must constantly identify funding sources to keep successful programs operating.

## CONCLUSIONS

Our results indicate that the UAB-CORD Summer Science Institute III program has had a positive impact on students pursuing education and careers in STEM fields. Providing experience in a research setting gave these students exposure to a realistic understanding of scientific discipline, experimental success and failure, hands-on training, and the opportunity to explore a STEM field. Mentors, including near-peer graduate students, play an important role in the success of the internship program by fostering an understanding of their scientific discipline, encouraging critical thinking and problem-solving, and teaching technical skills. SSI-III and other internship programs have increased the diversity of students entering STEM education and careers.

## Figures and Tables

**Table 1.** UAB-CORD SSI III Program Pre/Post Statistics for High School Students (N = 10). Students retrospectively recorded pre/post-participation ratings (0 = not at all, 1 = low, 2 = moderate and 3 = high) of their **skills, abilities, interest,** and **confidence**. The paired t test was used to test for pre/post mean differences at the 0.0500-level of significance with null hypothesis: difference = 0 and alternative hypothesis: difference <>0. Mean rating ($\bar{x}$), standard deviation (SD), matched pair dependent t-statistic (t), p-value (p), the correlation between the matched pair ratings (r), mean rating ($\bar{x}$), standard deviation (SD), matched pair dependent t-statistic (t), p-value (p), the correlation between the matched pair ratings (r), and effect size are displayed.

Item	Pretest		Posttest		r	t	P	Effect Size
	$\bar{x}$	SD	$\bar{x}$	SD				
Your confidence in putting a research poster together	1.40	0.97	2.60	0.52	0.17	4.81	0.0010	1.96 Large
Your ability to write a report using formal academic writing	1.90	0.74	2.70	0.48	−0.09	3.21	0.0107	1.50 Large
Your skills for preparing and presenting a science topic to your peers	1.90	0.74	2.70	0.48	0.53	4.00	0.0031	1.23 Large
Your ability to work independently	2.30	0.67	2.60	0.70	0.75	1.96	0.0811	0.44 Small
Your ability to work as a member of a team	2.40	0.52	2.90	0.32	0.27	3.00	0.0150	1.15 Large
Your skills for solving problems that arise during research of other hands-on projects	2.10	0.99	2.80	0.42	0.85	3.28	0.0095	0.57 Medium
Your interest in a career in a science field	2.60	0.52	3.00	0.00	Cannot calculate	2.45	0.0368	Cannot calculate
Your interest in a career that includes research	1.90	0.74	2.60	0.70	0.77	4.58	0.0013	0.98 Large

## ABBREVIATIONS

CORD: Center for Community OutReach Development
ELISA: Enzyme-Linked Immunosorbent Assay
PCR: Polymerase Chain Reaction
SSS: Summer Science Institute
STEM: Science, Technology, Engineering, and Mathematics
UAB: University of Alabama at Birmingham
US: United States
